# 肺结节患者焦虑抑郁与Th17/Treg和炎症水平改变的相关性研究

**DOI:** 10.3779/j.issn.1009-3419.2020.102.30

**Published:** 2020-07-20

**Authors:** 丽娜 王, 媛媛 韦, 华青 胡, 晓雨 张, 美娟 郑, 广鹤 费

**Affiliations:** 1 230022 合肥，安徽医科大学第一附属医院呼吸与危重症医学科 Department of Respiratory and Critical Care Medicine, The First Affiliated Hospital of Anhui Medical University, Hefei 230022, China; 2 230022 合肥，安徽省胸科医院介入肺脏病科 Department of Interventional Pulmonary Disease, Anhui Chest Hospital, Hefei 230022, China; 3 230022 合肥，安徽医科大学第一附属医院健康体检中心 Department of Health Examination Center, The First Affiliated Hospital of Anhui Medical University, Hefei 230022, China; 4 230022 合肥，安徽医科大学第一附属医院检验科 Department of Clinical Laboratory, The First Affiliated Hospital of Anhui Medical University, Hefei 230022, China

**Keywords:** 肺结节, 焦虑, 抑郁, 免疫细胞, 炎症, Pulmonary nodules, Anxiety, Depression, Immunity cellular, Inflammation

## Abstract

**背景与目的:**

肺癌的发病率逐年攀升，人们在体检时尤其注重对肺进行检查，得益于低剂量计算机断层扫描技术的普及，不仅肺癌能够得到早期诊断，很多肺结节也在体检时被发现，并成为当下不可忽视的重要健康问题。肺结节患者极易出现焦虑、抑郁等不良情绪，研究表明情绪障碍患者存在机体免疫功能失调和炎症水平变化，本研究旨在探讨肺结节患者焦虑抑郁与Th17/Treg和炎症水平改变的相关性。

**方法:**

纳入2019年4月-2019年7月就诊于安徽医科大学第一附属医院门诊受试者共143例，采用贝克焦虑量表（Beck Anxiety Inventory, BAI）、贝克抑郁量表第二版（Beck Depression Inventory-Ⅱ, BDI-Ⅱ）进行评估。其中健康对照（healthy control, HC）组40例；肺结节患者103例，依据量表评分分为焦虑和（或）抑郁（anxiety and/or depression, AD）组41例和无焦虑抑郁（non-anxiety and non-depression, NAD）组62例。检测外周血辅助性T细胞17（T helper cell 17, Th17）、调节T细胞（regulatory T cells, Tregs）百分比，计算Th17细胞绝对值；检测血清炎症因子，并分析组间差异及相关性。

**结果:**

三组间Th17细胞百分比、Th17细胞绝对数、Th17/Treg、白细胞介素-2（interleukin-2, IL-2）、IL-6、肿瘤坏死因子-α（tumor necrosis factor-α, TNF-α）水平的差异有统计学意义（均*P* < 0.001），AD组高于HC组和NAD组（均*P* < 0.05），HC组和NAD组间差异无统计学意义（均*P* > 0.05）。以上指标与焦虑、抑郁的严重程度无显著相关性（*P* > 0.05）。Tregs百分比、IL-4、IL-10水平在各组间差异无统计学意义（均*P* > 0.05）。女性肺结节患者焦虑和（或）抑郁比例高于男性（*P* < 0.05）。

**结论:**

肺结节患者易出现不同程度的焦虑、抑郁的情绪变化，进而导致机体出现免疫功能失调和低级别炎症。

## Abstract (en)

**Background and objective:**

The incidence of lung cancer is increasing annually. Clinicians pay special attention to lung tests during physical examinations. Due to the popularity of low-dose computed tomography, not only can lung cancer be diagnosed early, but physical examinations often reveal the presence of pulmonary nodules, an important health issue that cannot be ignored. Patients with pulmonary nodules are prone to adverse emotions such as anxiety and depression. Many studies have shown that patients with emotional disorders have immune system dysfunction and changes in inflammation levels. This study aimed to investigate the changes in anxiety, depression, the ratios of T helper cells 17 (Th17) and regulatory T cells (Tregs), and inflammation levels in patients with pulmonary nodules.

**Methods:**

A total of 143 subjects from The First Affiliated Hospital of Anhui Medical University were included from April 2019 to July 2019. All of the subjects were assessed with the Beck Anxiety Inventory (BAI) and the Beck Depression Inventory-Ⅱ (BDI-Ⅱ). Overall, 40 cases were healthy controls (HC) and 103 cases were patients with pulmonary nodules. The patients were divided into two groups according to the scale scores: 62 cases in a non-anxiety and non-depression (NAD) group and 41 cases in an anxiety and/or depression (AD) group. The percentage of Th17 and Tregs in the peripheral blood and inflammatory factors in the serum were detected. The absolute Th17 cell counts were calculated and the differences between the groups and correlations between these indicators were analyzed.

**Results:**

There were statistically significant differences in the percentage of Th17 cells, the absolute counts of Th17 and Th17/Treg cells, and the levels of interleukin-2 (IL-2), IL-6, and tumor necrosis factor-α (TNF-α) among three groups (all*P* < 0.001). The AD group was higher than the HC and NAD groups (all *P* < 0.05). There was no statistically significant difference between the HC and NAD groups (all *P* > 0.05). The previously described indicators had no significant correlation with the severity of anxiety and depression (*P* > 0.05). There were no significant differences in the percentage of Tregs or levels of IL-4 and IL-10 between the groups (all *P* > 0.05). The proportion of anxiety and/or depression in female patients with pulmonary nodules was higher than that in males (*P* < 0.05).

**Conclusion:**

Patients with pulmonary nodules are prone to varying degrees of anxiety and depression, which leads to immune dysfunction and low-grade inflammation.

肺结节是指影像学表现为肺周围孤立或多发的直径≤3 cm，局灶性、类圆形的实性或亚实性密度增高影，不伴有肺不张、肺门淋巴结肿大和胸腔积液^[1]^。由于人口老龄化、环境变化和空气污染等原因，肺结节的发病率日益增高，且得益于胸部低剂量计算机断层扫描（low-dose computed tomography, LDCT）的普及^[2]^以及大众对健康体检的意识逐渐提高，越来越多的肺结节被发现。虽然大多数肺结节是良性的，但近半数的肺结节患者由于缺乏对疾病的正确认识，产生焦虑、抑郁等不良情绪变化^[3]^。来源于生理和心理上的压力能激活包括辅助性T细胞17（T helper cell 17, Th17）、调节T细胞（regulatory T cells, Tregs）在内的神经系统中的免疫细胞，诱导促炎细胞因子的释放，从而导致神经递质改变和行为改变，并且这种现象在成年女性中表现的更为显著^[4-6]^。Th17细胞和Tregs源自共同的幼稚T细胞前体，Th17细胞是在转化生长因子-β（transforming growth factor-β, TGF-β）和白细胞介素（interleukin, IL）-6等细胞因子协同作用下产生的介导炎症反应的免疫细胞，Tregs是由CD4^+^ T淋巴细胞在TGF-β等细胞因子协同作用诱导下产生的，能够抑制机体过强的免疫反应的一种重要的免疫细胞。正常情况下，Th17细胞和Tregs保持动态平衡，二者失衡将会导致异常的免疫应答^[7]^。目前的研究主要是采用影像学方法对肺结节进行早期诊断和良恶性的判别，而患者的不良情绪以及可能由此导致的不良生理及临床后果并未引起足够重视。本研究评估了肺结节患者的焦虑和抑郁状态，检测外周血Th17细胞、Tregs百分比及炎症因子等，并探讨其相互关系及临床价值。

## 1 资料与方法

### 1.1 研究对象

纳入2019年4月-2019年7月于安徽医科大学第一附属医院门诊就诊的肺结节患者103例；纳入同期本院体检中心健康体检者40例组成健康对照（healthy control, HC）组。HC组入组标准：①贝克焦虑量表（Beck Anxiety Inventory, BAI） < 10分且贝克抑郁量表第二版（Beck Depression Inventory-Ⅱ, BDI-Ⅱ） < 14分；②胸部LDCT明确无肺结节及其他肺部疾病；③无精神、神经、免疫系统及其他慢性疾病病史；④体检未发现其他系统疾病；⑤2周内未服用任何药物。肺结节组纳入标准：①经胸部LDCT发现肺结节时间≤3个月的首次正规就诊患者；②一般情况良好，能够完善相关检查及心理测评；③年龄≥18岁。排除标准：①弥漫性肺结节（肺结节数量 > 10个）；②随访6个月，排除肺结节明显变化疑有恶变可能，或行手术治疗后病理结果为恶性者；③合并精神、神经系统、免疫系统疾病及其他慢性疾病；④合并恶性肿瘤；⑤2周内发生过感染；⑥2周内使用过抗炎、抗过敏、糖皮质激素、免疫抑制剂等药物。本研究经安徽医科大学生物医学伦理委员会批准，所有受试者均签署知情同意书。

### 1.2 实验室器材和试剂

美国BD公司FACSCanto Plus流式细胞仪；BD Bioscience公司FITC CD4、PE CD25、APC FoxP3、PE IL-17A、PE IgG1单克隆抗体、人细胞因子检测试剂盒；eBioscience公司穿膜液、固定液。

### 1.3 情绪状态评估

所有研究对象均采用BAI、BDI-Ⅱ量表进行情绪状态测评（肺结节患者在我院首次就诊时进行）。BAI < 10分为无焦虑，10分-18分为轻-中度焦虑、19分-29分为中-重度焦虑、30分-63分为重度焦虑；BDI-Ⅱ评分 < 14分为无抑郁，14分-19分为轻度抑郁，20分-28分为中度抑郁，29分-63分为重度抑郁^[8, 9]^。据此将103例肺结节患者分为无焦虑抑郁（non-anxiety and non-depression, NAD）组、焦虑和（或）抑郁（anxiety and depression, AD）组。

### 1.4 标本采集

所有受试者在进行情绪评估的当天上午9时-11时采集空腹静脉血2 mL于EDTA抗凝管中，用于血常规及流式细胞术检测；采集静脉血5 mL于促凝管采集中，3, 500 rpm离心5 min后，取血清保存于-80 oC冰箱备用。

### 1.5 流式细胞术检测Th17、Treg细胞

抗凝血与磷酸盐缓冲液（phosphate buffer saline, PBS）1:1稀释混匀，取淋巴细胞分离液置于离心管中，倾斜管体沿管壁缓慢加入稀释血液。20 oC以800 *g*离心30 min，吸出中间白膜层即外周血单个核细胞（peripheral blood mononuclear cell, PBMC）置于流式管，PBS洗涤、稀释、涡旋混匀后，均分于2支流式管，分别标注Tregs及Th17，另外随机留取一份PBMC作为Th17细胞对照。Tregs管加入CD4、CD25单抗孵育、固定、穿膜后加入FoxP3单抗孵育，洗涤、PBS混匀上机检测。Th17管及对照管分别加入由DMEM、小牛血清、离子霉素、莫能菌素、豆蔻酰佛波醇乙酯、青链霉素双抗配制的培养基，置于37 oC温箱孵育5 h。后加入CD4单抗孵育、固定、穿膜。Th17管加入IL-17A单抗、对照管加入IgG1单抗孵育，洗涤、PBS混匀上机检测。采用FlowJo V10.0.1软件进行数据分析。利用血常规中淋巴细胞绝对数计算Th17细胞绝对数。

### 1.6 多重液相蛋白定量技术（cytometric bead array, CBA）检测炎症因子

按照CBA试剂盒说明书制备细胞因子标准品，利用标准品梯度稀释以配制标准曲线；分别取IL-2、IL-4、IL-6、IL-10、肿瘤坏死因子-α（tumor necrosis factor-α, TNF-α）捕获磁珠混匀，离心去上清后加入血清刺激液避光孵育。标椎曲线中加入混合磁珠、抗体，待测标本中加入混合磁珠、血清、抗体，混匀避光孵育3 h，洗涤后加入PBS上机检测。采用BD FACSDiva V6.0软件进行数据分析。

### 1.7 统计学方法

采用SPSS 23.0软件进行统计分析，符合正态分布的计量资料采用均数±标准差（Mean±SD）表示，不符合正态分布的资料采用中位数（M）和四分位数间距（interquartile range, IQR）表示，各组间年龄、体重指数（body mass index, BMI）、免疫细胞、炎症因子的比较采用单因素方差分析；各组间性别构成、吸烟人数以及性别对患者焦虑抑郁状态的影响采用*χ*^2^检验；各组间受教育年限、BAI总分、BDI-Ⅱ总分的比较采用*Kruskal-Walls H*检验；AD组中免疫细胞、炎症因子与焦虑、抑郁程度采用*Spearman*秩相关分析，以*P* < 0.05为差异有统计学意义。

## 2 结果

### 2.1 两组一般资料比较

根据入组和排除标准，共143例受试者纳入本研究，HC组40例，NAD组62例、AD组41例，各组间年龄、性别、吸烟人数、BMI、受教育年限差异均无统计学意义（*P* > 0.05），见表 1。

**1:** 各组受试者的一般临床资料 General clinical data of subjects in each group

Items	HC (*n*=40)	NAD (*n*=62)	AD (*n*=41)	Statistic value	*P*
Age (yr) (Mean±SD)	49.7±12.8	53.3±11.0	47.6±13.8	2.754^a^	0.067
Gender (Male/Female) (*n*)	22/18	39/23	17/24	4.580^b^	0.101
Population of smoking (*n*)	3	8	1	3.367^b^	0.155
BMI (Mean±SD, kg/m^2^)	23.6±2.7	23.4±2.9	23.9±3.0	0.792^a^	0.455
Education (yr) [M (IQR)]	15.0 (4.0%)	15.0 (7.0%)	15.0 (7.0%)	3.087^b^	0.214
BAI [M (IQR)]	2.5 (3.0%)	2.0 (3.0%)	11.0 (5.0%)	80.527^b^	< 0.001
BDI-Ⅱ [M (IQR)]	4.5 (5.0%)	5.0 (4.3%)	12.0 (7.0%)	66.774^b^	< 0.001
HC: healthy control; NAD: non-anxiety and non-depression; AD: anxiety and/or depression; BMI: body mass index; BAI: Beck Anxiety Inventory; BDI-Ⅱ: Beck Depression Inventory-Ⅱ. a: F value; b: *χ*^2^ value.					

### 2.2 不同性别肺结节患者焦虑/抑郁比较

女性肺结节患者47例，其中无焦虑抑郁23例，焦虑和（或）抑郁24例；男性肺结节患者56例，其中无焦虑抑郁39例，焦虑和（或）抑郁17例，差异有统计学意义（*χ*^2^=4.573, *P*=0.032）。

### 2.3 组间Th17细胞百分比、Th17细胞绝对数、Th17/Treg、IL-2、IL-6、TNF-α水平比较

Th17细胞百分比、Th17细胞绝对数、Th17/Treg、IL-2、IL-6、TNF-α水平在HC组、NAD组、AD组间的差异有统计学意义（均*P* < 0.001），AD组要高于HC组和NAD组；HC组和NAD组间的差异无统计学意义（均*P* > 0.05）。Tregs百分比、IL-4、IL-10水平在各组间差异无统计学意义（均*P* > 0.05）（表 2，图 1）。

**2:** 各组受试者免疫细胞和炎症因子的统计学结果（Mean±SD） Statistical results of immune cells and inflammatory factors of subjects in each group (Mean±SD)

Immune cells and inflammatory cytokines	HC (*n*=40)	NAD (*n*=62)	AD (*n*=36)	*F*	*P*
Th17 cells (%)	1.45±0.59	1.61±0.65	2.37±0.68	24.549	< 0.001
Tregs (%)	6.31±1.64	6.22±1.48	5.53±1.54	3.239	0.142
Absolute counts of Th17 (×10^7^/L)	2.84±1.47	3.18±1.77	4.56±1.60	13.008	< 0.001
Th17/Treg	0.25±0.14	0.26±0.09	0.46±0.18	32.860	< 0.001
IL-2 (pg/mL)	1.41±0.36	1.42±0.36	2.04±0.17	23.971	< 0.001
IL-4 (pg/mL)	1.78±0.69	1.95±0.74	1.99±0.79	0.884	0.415
IL-6 (pg/mL)	3.15±1.69	3.43±1.70	5.27±2.14	16.697	< 0.001
IL-10 (pg/mL)	2.42±1.09	2.82±1.13	2.56±0.84	1.973	0.143
TNF-α (pg/mL)	5.78±2.97	6.31±3.11	10.33±4.93	19.429	< 0.001
IL: interleukin; TNF-α: tumor necrosis factor-α; Th17: T helper cell 17; Tregs: regulatory T cells.					

**1:**
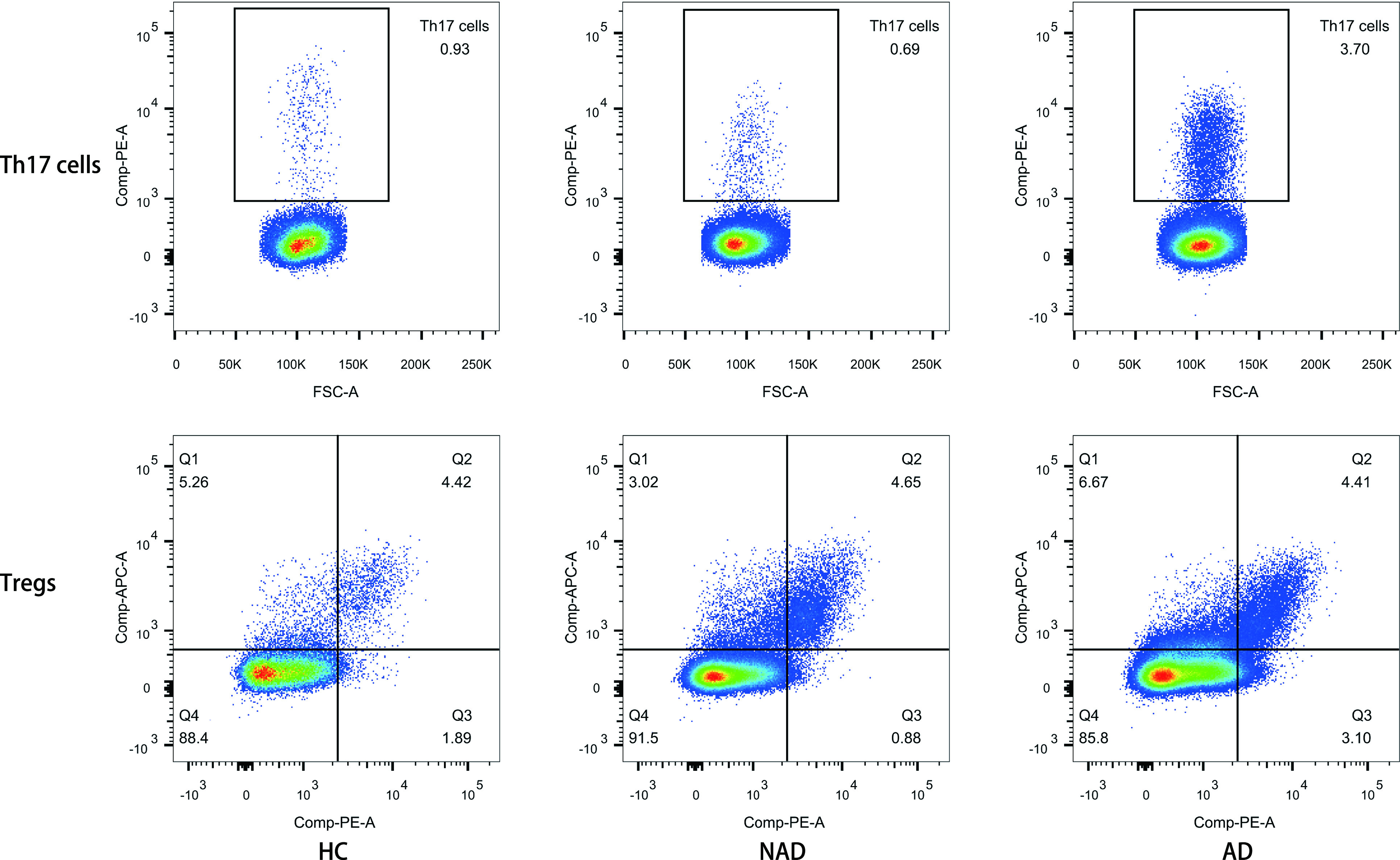
三组间Th17细胞和Tregs百分比。相比于HC组及NAD组，AD组的Th17细胞百分明显升高（*P* *<* 0.001），而HC组与NAD组之间无显著差异（*P*=0.237）。Tregs百分比在三组间的差异无统计学意义（*P*=0.142）。 The percentage of Th17 cells and Tregs among three groups. The percentage of Th17 cells in AD group was higher than the HC and NAD groups (*P* < 0.001), while there was no significant difference between HC group and NAD group (*P*=0.237). There were no statistically significant differences in the percentage of Tregs among three groups (*P*=0.142).

### 2.4 焦虑/抑郁相关因素分析

AD组中无焦虑5例，轻-中度焦虑31例，中-重度焦虑4例，重度焦虑1例；患者焦虑程度与Th17细胞百分比（*r_s_*=-0.271）、Th17细胞绝对数（*r*=-0.189）、Th17/Treg（*r_s_*=-0.024）、IL-2（*r_s_*=-0.284）、IL-6（*r_s_*=-0.068）、TNF-α（*r_s_*=-0.036）水平无显著相关性（*P* > 0.05）。无抑郁23例，轻度抑郁14例，中度抑郁2例，重度抑郁2例；患者抑郁程度与Th17细胞百分比（*r_s_*=0.131）、Th17细胞绝对数（*r_s_*=0.247）、Th17/Treg（*r_s_*=-0.039）、IL-2（*r_s_*=-0.014）、IL-6（*r_s_*=0.051）、TNF-α（*r_s_*=-0.097）水平无显著相关性（*P* > 0.05）。

## 3 讨论

近年来，人们健康意识日益增强，由LDCT筛查出的肺结节越来越多，这虽然提高了早期肺癌的诊断率，但同时也给一些患者带来了严重的心理负担，导致反复多处就诊等过度医疗行为。世界卫生组织关于精神疾病的调查结果显示我国成年人焦虑障碍和抑郁障碍的患病率分别为4.8%和3.6%，这一比例在本研究的肺结节患者中分别为35%和17%^[10]^，明显高于大众人群焦虑、抑郁障碍的患病率，说明肺结节的确会使许多患者产生精神压力。此外，在本研究的实际调查中还发现处于焦虑抑郁状态的肺结节患者不仅有紧张、恐惧、情绪低落等情感变化，在发现肺结节后还会出现胸闷、胸痛、头晕等与肺结节无关的症状，甚至出现食欲、睡眠、性欲等的改变，这极大地影响了患者的日常生活。已有研究^[5]^观察到焦虑和抑郁障碍的患者存在免疫细胞亚群的失衡和促炎细胞因子水平的升高，但在出现焦虑、抑郁的肺结节患者中是否也存在这一现象仍不得而知。

BAI是一种简便易行的焦虑测评量表，与其他常用的量表如汉密尔顿焦虑量表、状态-特质焦虑问卷相比，同样有着良好的效度^[11]^。BAI的特点是能够排除抑郁的干扰而获得较准确的焦虑评分，因此已被广泛地用于多种疾病状态下的心理评估。不仅如此，BAI还具有时间敏感性，尤其适用于对肺结节患者焦虑状态的长期随访下的评价^[12]^。BDI-Ⅱ在区分抑郁和非抑郁受试者上有着极高的可靠性，是衡量抑郁症严重程度的一种经济有效的量表，目前已使用超过22年，被翻译成近20种语言，在全球逾7, 000项的研究和临床实践中被广泛使用^[13]^。因此本研究选择BAI和BDI-Ⅱ来评估肺结节患者是否处于焦虑、抑郁的状态，并且通过流式细胞和CBA技术来检测受试者的免疫细胞及炎症因子水平，进而探讨患者是否因情绪的变化导致机体出现免疫失调及炎症因子水平的改变。

由于血清免疫细胞、炎症因子易受多种因素的影响，尤其是Th17与Treg在肺癌的免疫调节中发挥了重要的作用^[14]^，因此排除肺癌患者对提高本研究的准确性至关重要。显然，对全部肺结节患者都进行病理学检查以明确性质并不现实，所以本研究通过对患者进行连续6个月的随访尽可能地排除肺癌。此外，本研究还排除了神经精神疾病、感染、肿瘤和心血管系统、内分泌系统、免疫系统疾病及服用各种药物的情况。

本研究发现，相比于HC组和NAD组，AD组患者的外周血Th17细胞百分比、Th17细胞绝对值、Th17/Treg及IL-2、IL-6、TNF-α水平显著升高。Th17细胞百分比、Th17细胞绝对值、Th17/Treg的升高提示机体免疫功能出现失调，IL-2、IL-6、TNF-α水平的升高则提示机体存在异常的炎症反应。而上述指标在HC组及NAD组间差异无统计学意义，这说明免疫细胞及炎症因子水平的改变与焦虑、抑郁障碍密切相关，而非肺结节本身所造成。Bekhbat等^[5]^的研究发现在经历几小时到几周的持续压力作用下，机体生理上可能发生永久改变，进而干扰免疫系统的正常功能，导致炎症或疾病状态的发生。很多疾病都可能共患焦虑、抑郁，这些患者暴露于创伤和压力下会导致下丘脑-垂体-肾上腺（hypothalamic-pituitary-adrenal, HPA）轴的敏感性提高、机体免疫系统激活，并释放促炎细胞因子^[15]^，随着压力暴露时间的延长，HAP和免疫系统相继出现功能紊乱^[16]^，导致免疫功能失调以及异常炎症反应。这解释了多种免疫细胞和炎症因子在肺结节合并焦虑和抑郁的患者中异常升高的现象。然而情绪是十分复杂的高级认知活动，情绪障碍的时长、抑制炎症通路的激活以及生活环境等因素的差异可能是导致本研究中焦虑组和抑郁组患者免疫细胞和炎症因子水平的变化与焦虑、抑郁程度无相关性的原因。

虽然许多研究表明在精神障碍疾病中，促炎细胞因子（如IL-1β、IL-2、IL-6、TNF-α、IFN-γ、IL-17等）水平显著升高^[17, 18]^，但抗炎因子水平的变化并不一致。Zou等^[19]^发现抑郁症患者抗炎细胞因子水平升高，而Wang等^[20]^却发现创伤后应激障碍患者的抗炎因子水平正常。本研究中各组间IL-4、IL-10水平差异无统计学意义，这提示抗炎因子对精神障碍的作用机制暂不明确，且易受多种因素的影响。

Faravelli等^[4]^发现在同样压力下，女性患焦虑抑郁的概率是男性的1.8倍，而本研究发现，肺结节患者中出现焦虑抑郁的男女比例为1:1.7，Slatore等^[3]^的研究表明39%的患者因偶然发现肺结节感到不同程度的心理压力，在医生与患者充分的沟通后可以减轻心理压力所带来的困扰。这提示在临床工作中需要对肺结节患者尤其是女性患者的心理状态予以更多的关注，指导科学治疗。

肺结节患者出现情绪障碍，主要是由于缺乏对疾病的正确认识，将肺结节直接等同于肺癌。本研究证实这种情绪状态会导致免疫功能失调和低级别炎症。病理生理学研究^[16]^发现情绪障碍与机体炎症、免疫功能失调间存在相互促进作用：免疫失调和炎症会使患者产生焦虑、抑郁等情绪障碍，而情绪障碍会进一步加重这一过程的进展，因此如果不及时地对患者进行心理疏导，给予科学合理的治疗建议，不仅会加重患者的情绪障碍，而且可能会影响到疾病的预后。早期发现并及时给予心理干预，调节患者的情绪状态，将极大地改善患者的依从性，阻断炎症、免疫失调和不良情绪间的恶性循环，提高肺结节治疗的规范性和有效率。

本研究的局限性在于病例数较少，且未能研究心理干预治疗是否会对相关指标的好转及肺结节的预后产生影响。此外，患者入组时均系近期发现的肺结节，因此暂未能获得长期随访资料，而患者的心理状态是会随着时间不断变化的。但本项研究还在继续，入组的患者数量不断增加，对患者的随访尤其是病理结果的跟踪也在持续进行。相信通过对免疫细胞、炎症指标和心理状态的动态观测，以及加入心理干预的治疗研究，将会得到更有价值和深度的研究结果，从而更好地指导临床治疗。
